# Innovative Animal Model of DSS-Induced Ulcerative Colitis in Pseudo Germ-Free Mice

**DOI:** 10.3390/cells9122571

**Published:** 2020-12-01

**Authors:** Sona Gancarcikova, Stanislav Lauko, Gabriela Hrckova, Zuzana Andrejcakova, Vanda Hajduckova, Marian Madar, Livia Kolesar Fecskeova, Dagmar Mudronova, Kristina Mravcova, Gabriela Strkolcova, Radomira Nemcova, Jana Kacirova, Andrea Staskova, Stefan Vilcek, Alojz Bomba

**Affiliations:** 1 Department of Microbiology and Immunology, University of Veterinary Medicine and Pharmacy in Kosice, 041 81 Kosice, Slovakia; sona.gancarcikova@uvlf.sk (S.G.); vanda.hajduckova@uvlf.sk (V.H.); marian.madar@uvlf.sk (M.M.); dagmar.mudronova@uvlf.sk (D.M.); radomira.nemcova@uvlf.sk (R.N.); jana.kacirova@student.uvlf.sk (J.K.); 2 Institute of Parasitology, Slovak Academy of Sciences, 041 81 Kosice, Slovakia; hrcka@saske.sk; 3 Department of Biology and Physiology, University of Veterinary Medicine and Pharmacy in Kosice, 041 81 Kosice, Slovakia; zuzana.andrejcakova@uvlf.sk; 4 Institute of Microbiology, Czech Academy of Sciences, Centre Algatech, 379 01 Trebon, Czech Republic; livia.fecskeova@gmail.com; 5 Department of Epizootology, Parasitology and Protection of One Health, University of Veterinary Medicine and Pharmacy in Kosice, 041 81 Kosice, Slovakia; kristina.mravcova@student.uvlf.sk (K.M.); gabriela.strkolcova@uvlf.sk (G.S.); Stefan.Vilcek@uvlf.sk (S.V.); 6 Department of Stomatology and Maxilofacial Surgery, Pavol Jozef Safarik University in Kosice, 041 81 Kosice, Slovakia; andrea.staskova@upjs.sk; 7 Institute of Experimental Medicine, Faculty of Medicine, Pavol Jozef Safarik University in Kosice, 041 81 Kosice, Slovakia; alojz.bomba@upjs.sk

**Keywords:** pseudo germ-free model, antibiotics, DSS-induced colitis, gut microbiota, histopathology

## Abstract

The aim of this study was to investigate the use of a standardized animal model subjected to antibiotic treatment, and the effects of this treatment on the course of dextran sodium sulphate (DSS)-induced colitis in mice. By decontamination with selective antibiotics and observation of pathogenesis of ulcerative colitis (UC) induced chemically by exposure of mice to various concentrations of DSS, we obtained an optimum animal PGF model of acute UC manifested by mucin depletion, epithelial degeneration and necrosis, leading to the disappearance of epithelial cells, infiltration of *lamina propria* and submucosa with neutrophils, cryptitis, and accompanied by decreased viability of intestinal microbiota, loss of body weight, dehydration, moderate rectal bleeding, and a decrease in the selected markers of cellular proliferation and apoptosis. The obtained PGF model did not exhibit changes that could contribute to inflammation by means of alteration of the metabolic status and the induced dysbiosis did not serve as a bearer of pathogenic microorganisms participating in development of ulcerative colitis. The inflammatory process was induced particularly by exposure to DSS and its toxic action on compactness and integrity of mucosal barrier in the large intestine. This offers new possibilities of the use of this animal model in studies with or without participation of pathogenic microbiota in IBD pathogenesis.

## 1. Introduction

Intestinal microbiota is the largest and most diverse community of microorganisms in the human body. The symbiotic relationship between intestinal microbiota and the host is regulated and stabilized by a complex network of interactions that include metabolic, immune, and neuroendocrine relationships among them [1]. The pathologically unimpaired intestinal microbiota acts symbiotically, produces vitamins, suppresses expansion of pathological microorganisms and facilitates digestion of food substrates while constantly interacts with the immune system of the host. The optimum composition of healthy gut microbiota differs among individuals. The more abundant and diverse the microbiota turns in the course of life the better the organism will resist to attacks from the outer environment. Indeed, intestinal microbiota constitutes a changing ecosystem considerably burdened by many factors, such as unbalanced food, stress, use of antibiotics, or suffering from diseases [2]. Impairment of this delicate ecosystem between the host and the microbiota may interfere with the development of the immune system. Its adverse quantitative and qualitative changes referred to as dysbiosis result in the development of pathological states [3,4,5,6,7]. The role of intestinal microbiota is still insufficiently understood, however, without any doubts there exists a tight relationship between dysbiosis of gut microbiota and disorders of other organ systems. This is the reason why dysbiosis can be considered a biomarker of such disorders and the study of balance in the intestinal microbiota is one of the priorities of therapy of diseases [5].

The term idiopathic inflammatory bowel diseases (IBD) refers to a group of chronic diseases of the digestive tract characterized by alternating periods of relapse and remissions based on complex interactions between genetic predisposition of an individual, intestinal microbiota, and the immune system [8]. The disease is associated with intestinal microbial dysbiosis including expansion of facultative anaerobic bacteria in the family Enterobacteriaceae [9]. Crohn’s disease (CD) and ulcerative colitis (UC) are two main manifestations of IBD. These two forms of IBD differ particularly by localization of inflammatory foci in the digestive tract and the extent of histopathological changes in the intestinal wall [10,11]. Induction and subsequent progression of the intestinal inflammation are the consequences of a complex multifactorial interaction between the host and his environment [12]. Animal models of IBD may help to obtain new knowledge and to understand the basic mechanisms of inflammatory diseases and can be useful in testing of innovative treatment approaches. Experimental induction of the UC model using gnotobiotic animals will enable systematic manipulation with the variable factors and allow scientists to verify or reject their presumptive hypotheses [13]. However, we have to face the problem that animals with germ-free (GF) microbiological status, i.e., without microbiota, have some specific properties. They differ from animals with conventional microbiota in various aspects including anatomical, immunological, and physiological parameters. Felenius et al. [14] and Kitajima et al. [15] observed in their studies development of an optimum model colitis induced by dextran sodium sulphate (DSS) in GF animals, i.e., at the absence of microbiota. On the other hand, other studies [16] reported only weak inflammatory response to DSS induced colitis in GF mice, or even pronounced bleeding with high mortality and absence of inflammatory changes in the intestinal mucosa of such animals [17]. Chirlaque et al. [18] concluded that absence of microbiota considerably reduces inflammation of large intestine after exposure to DSS but also impairs function of the intestinal barrier.

Induction of gut dysbiosis by means of *per os* administration of antibiotics facilitates elucidation of the importance of intestinal microbiota in pathogenesis of IBD. The antibiotic-treated or pseudo germ-free (PFG) mice are not completely free of bacterial load but its significant reduction results also in the changes in signaling pathways, morphology of organs, and response to antibiotic decontamination. The results are parallel with those observed in germ-free mice. However, while administration of antibiotics offers a cheap and available alternative to germ-free models, results obtained by nonstandardized procedures are heterogeneous as they are affected by acquired incomplete or inconsistently removed microbiota. For this reason, comparability of the studies based on administration of antibiotics is lower than that of studies on germ-free animals. 

In the studies by Popper et al. [19] and Gancarčíková et al. [20] decontamination of the SPF mice of BALB/c line were obtained by administration of amoxicillin *per os* and potentiated with potassium clavulanate at a dose of 387.11 mg/kg body weight and ciprofloxacin administered *s.c.* at a dose of 18.87 mg/kg body weight every 12 h for 5 days. This resulted in a decreased viability of microorganisms in feces and the cecal content and reduction of cultivable microorganisms in the feces. By day 3 of the study the counts of the latter declined below the recovery level and the mice microbiota was reduced to two detected cultivable species, namely *Escherichia coli* (GenBank KX086704) and *Enterococcus* spp. (GenBank KX086705). The 10-day convalescence of decontaminated animals under gnotobiotic conditions prevented restoration of species diversity of mice microbiota and sufficed to return the metabolic, hematological, and morphological values to the physiological range.

On the basis of our previous studies we decided to investigate the use of a standardized antibiotic treatment model with respect to its effects on the course of DSS-induced colitis in mice. In this study, after obtaining a PGF animal model supported by observation of viability of cecal microbiota, detected cultivable bacteria, molecular analysis of gut microbiota composition, and production of SCFAs, the following clinical, histological, immunohistochemical, and microbiological parameters were investigated to evaluate the effects of acute colitis induced by DSS using following criteria, Disease Activity Index (DAI) score, hematology parameters, Histological Activity Index (HAI), histoarchitectural changes by light microscopy, morphometric parameters, nuclear antigen marker of cell proliferation (PCNA), anti-apoptotic (Bcl-xL) marker, and viability of the cecal microbiota.

## 2. Materials and Methods

### 2.1. Ethics Declarations

The presented experiment with protocol number 4073/18-221/3, was approved by the State Veterinary and Food Administration of the Slovak Republic. The animals were handled and sacrificed in a humane manner in accordance with the guidelines established by the relevant Ethics Committee of the University of Veterinary Medicine and Pharmacy in Kosice.

### 2.2. Animals, Their Housing and Diet

The experiment was carried out on 126 specific pathogen-free (SPF) BALB/c female mice, (4 weeks old), from the breeding facility Velaz Ltd. (Prague, Czech Republic). Conventional SPF mice were transported in special transport containers to the accredited Laboratory of Gnotobiology, of the Institute of Microbiology and Gnotobiology, UVMP in Kosice, Slovakia (SK U 16016). Prior to placing the laboratory animals into a gnotobiotic rodent isolator THF 3315 (EHRET Labor-und Pharmatechnik GmbH Co.&KG, Emmendingen, Germany) for rodent breeding and a two-sleeve CBC breeding isolator (CBC, Ltd., Madison, WI, USA), the surface of the vessels was disinfected with 2% peracetic acid. This was followed by a thorough venting of the peracetic acid vapors after which the mice were moved into breeding polypropylene containers, 7–8 mice in each, of the following dimensions: length 365 mm, width 207 mm, height 140 mm. The laboratory animals were fed ad libitum with complete mixed irradiation-sterilized (25 kGy) feed intended for mice in ST-1 breeding (Velaz Ltd., Prague, Czech Republic). They had continuous access to autoclaved water in glass bottles. The diet contained (kg diet) 24% crude protein, 4.4% crude fiber, 3.4% crude fat, 6.8% ash, 1.8 g sodium, 11 g calcium, 7.2 g phosphorus, 0.38 mg selenium, and 20 mg copper (vitamin D 2200 IU, vitamin A 28000 IU, vitamin E 100 mg). The mice were housed on litter intended for barrier breeding Lignocel 3–4 S (JRS, Rosenberg, Germany), an environmentally friendly product based on wood fibers derived from renewable raw materials that allowed the mice to manipulate with this material. The air entering and leaving the isolator (type EHRET THF 3315) was filtered through round filtering cartridges HEPA, functioning as a single-way filters of class H13, according to EN 1822. A ventilation system Fantech (model F64230) was fastened to the lower part of the construction of the CBC isolator. The supply and exhaust air passed through ventilation ducts equipped with CBC filtration system consisting of HEPA filters (class H13) of cylindrical shape, 127 mm in diameter. The filtration units installed in isolators ensured a minimum of 10–15 exchanges of air per hour (h) at overpressure of 50–70 kPa and air flow 8–30 m^3^. All experimental materials, including distilled water, glass and metal materials were sterilized by autoclaving at 121 °C and pressure 1.3 MPa for 30 min. Cellulose wadding and other sanitary material was gamma-irradiated (Bioster, Veverska Bityska, Czech Republic). Both isolators were equipped with systems of sensors connected to the Meshlium device that collected at hourly intervals information about air temperature, relative humidity, and concentration of air pollutants (hydrogen sulphide, toluene, ethanol, ammonia, and hydrogen) and recorded these data in an internal database MySQL (IBM Slovakia Ltd., Kosice, Slovakia). The optimum relative humidity was maintained at 45–65% and the optimum temperature at 20–24 °C. The noise level declared by the manufacturer in the gnotobiotic unit reached approximately 45 dB. Illumination of the gnotobiotic isolator for mice was provided by outer neon lighting fixtures and by natural illumination that ensured regular rhythm of light and darkness.

### 2.3. Microbiological Testing

Closed gnotobiotic units were disinfected with 2% peracetic acid. After 24 h exposure, residual vapors of the disinfectant were removed by ventilation. By means of sterile swabs, the surface of mice, their rectum, walls, and other material in isolators were swabbed into saline in 3-day intervals. Routine microbiological control was carried out using TSA agar (Tryptic Soy Agar) with 5% defibrinated ram blood (BBL, Microbiology system, Cockeysville, MD, USA). Spare isolators of CBC type (CBC, Ltd., Madison, WI, USA) were prepared and used. They were equipped with medical materials, sampling material, feed and autoclaved water that was needed throughout the experiment, which minimized the opening of connecting tunnels of isolators for essential processes.

### 2.4. Antibiotic Treatment

Amoxicillin is a broad spectrum bactericidal antibiotic of penicillin type used in combination with clavulanic acid that inhibits beta-lactamases and synthesis of bacterial cell walls. The dose lethal to mice is 4526 mg/kg. In our study we used preparation Amoksiklav 2 × 457 mg/5 mL (Sandoz Pharmaceuticals, Ljubljana, Slovenia) at a dose of 0.2 mL *per os* every 12 h for 5 days (dose of effective ingredient was 387.11 mg/kg/mouse) which amounted to 12-fold lower dose than the lethal one.

Ciprofloxacin is a member of the group of fluorochinolone antibiotics. The mechanism of effect of these antibiotics consists in inhibition of DNA synthesis by blocking the enzyme responsible for blocking the coiling and supercoiling of strands of the bacterial nucleic acid during the G phase of the cell cycle. The dose of ciprofloxacin lethal to mice is 5000 mg/kg. In the experiment we used preparation *Ciprinol con infusione* 5 × 10 mL/100 mg (Krka d.d., Novo mesto, Slovenia), at a dose of 0.1 mL *s.c.* every 12 h for 5 days (dose of effective ingredient was 19.60 mg/kg/mouse) which amounted to 255-fold lower dose than the lethal one.

### 2.5. DSS-Induced Colitis

**Figure 1:**
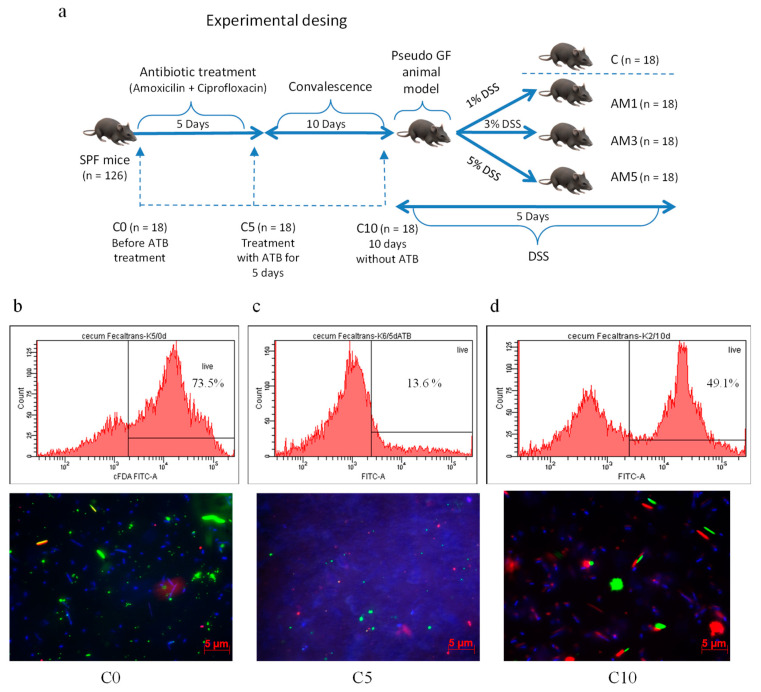
Experimental design and the viability of microorganisms in the cecum determined by Fluorescence-Activated Cell Sorting (FACS, histograms showing percentage of live bacteria based on intensity of green fluorescence (X axis) opposite to counts (Y axis); living bacteria emit high intensity of green fluorescence) and visualized with VFQTOPF (*VFQTOPF*—viability fluorescent quick test on a polycarbonate filter; live bacteria are green, dead are red, and barely active but not dead are blue). (**a**) Experimental design and the timeline. (**b**) Mice cecum before antibiotic treatment (C0). FACS analysis (73.5%) and VFQTOPF visualization. (**c**) Mice cecum on day 5 after ATB treatment (C5). FACS analysis (13.6%) and VFQTOPF visualization. (**d**) Mice cecum after 10 days following the termination of ATB treatment (C10). FACS analysis (49.1%) and VFQTOPF visualization.

Acute colitis in animals from experimental groups (AM1, AM3, AM5) was induced chemically by irradiated dextran sodium sulphate (DSS, molecular weight 40 kDa, TdB Consultancy AB, Upsala, Sweden) which was added daily in 1%, 3%, and 5% concentrations to autoclaved water supplied to animals in glass bottles for 5 days. The animals in control group (C) received autoclaved water only. After 10-day convalescence, the mice were divided into three experimental groups and one control group according to the scheme shown in Figure 1a. For the purpose of obtaining samples for microbiological, biochemical, and histological analysis, the PGF mice from individual groups were sacrificed humanely by administration of sodium pentobarbital at a dose of 86 mg/kg live weight with subsequent cervical dislocation on days 0, 5, 15, and 20, of the experiment.

### 2.6. Evaluation of Clinical Colitis

Our evaluation of clinical colitis included daily monitoring of disease activity score determined on the basis of stool consistency, blood in the stool, and weight loss during exposure to DSS. The relevant specific criteria that were used to calculate the DAI are presented in Table 1.

### 2.7. Hematological Analysis

Blood plasma was collected into tubes containing menadione ethylenediaminetetraacetic acid (K_3_EDTA) and was used for hematological analysis employing a BC-2008 VET automatic analyzer (Mindray, Shenzhen, China).

### 2.8. Short Chain Fatty Acid (SCFA) Analysis

Cecum (0.5 g) was diluted in 25 mL of deionized water, homogenized (stomacher; IUL Instruments), and filtered through a filter paper. A 30 μL aliquot was used for analysis of SCFAs (acetoacetic, lactic, succinic, acetic, propionic, butyric, valeric acids) by capillary isotachophoresis (Electrophoretic analyzer EA 202M, VILLA LABECO spol. s.r.o., Spisska Nova Ves, Slovakia). A leading electrolyte of the following composition was used in the pre-separatory capillary: 10 mmol/L HCl + 22 mmol/L ε-aminocaproic acid + 0.1% methylhydroxyethylcellulosic acid, pH 4.3. A solution of 5 mmol/L caproic acid + 20 mol/L histidine was used as a finishing electrolyte. This electrolytic system worked at 140 μA in the pre-separatory and at 40 μA in the analytic capillary.

### 2.9. Microbiological Cultivation

Samples for microbial analysis (feces, cecum) were homogenized in a Stomacher Lab Blender 80 (Seward Medical Limited, London, UK). Microbial populations in feces, cecum, and gingival plaque samples were determined according to the standard microbiological method using serial dilutions from 10^−1^ to 10^−7^. Bacterial strains were cultivated on TSA agar with 5% of defibrinated ram blood (BD MS, Cockeysville, MD, USA) and on Schaedler agar (HiMedia Laboratories, Mumbai, India) in a thermostat under aerobic and anaerobic conditions (BBL GasPak™ Plus, BD, MD, USA) at 37 °C for 24 h at aerobic and 48 h at anaerobic cultivation. The viable counts are presented as the log 10 of colony forming units (CFU) per gram of sample. The results are presented as arithmetical means ± standard deviation (SD).

### 2.10. Viability of Microorganisms in the Cecum Determined by Fluorescence-Activated Cell Sorting (FACS) Visualized with Viability Fluorescent Quick Test on a Polycarbonate Filter (VFQTOPF)

The viability of microorganisms in the cecum of mice was determined after simultaneous staining of samples with carboxyfluorescein diacetate and propidium iodide using a BD FACS Canto flow cytometer (Becton Dickinson and Company, NJ, USA) and Carl Zeiss Axio Observer Z1 epifluorescence microscope. Axio Vision Rel 4.8 software was used for the microphotography analysis. The methods were performed according Gancarčíková et al. [20].

### 2.11. Identification of Cultivable Bacteria Based on PCR and Their Sequences of 16S rRNA Gene

Solitary colonies grown on MRS broth (Merck, Darmstadt, Germany) and on TSA agar with 5% ram’s blood (BBL, Microbiology systems, Cockeysville, MD, USA) were used as a source for DNA isolation. DNA was isolated by DNAzol direct (Molecular Research Center Inc., Cincinnati, OH, USA) according manufacturer’s instructions. Low Bind Eppendorf tubes and DNA RNA free tips with filters (Greiner Bio-One, Frickenhausen, Germany) were used for processing. The PCR conditions were as follows: 5 min hot start at 94 °C; 31 cycles of 1 min at 94 °C, 1 min at 55 °C, and 3 min at 72 °C; a final extension step of 10 min at 72 °C. The PCR was set up on parameters for one sample to total volume of 50 µL [containing 25 µL Mastermix 2XMM One Taq Mastermix (New Engladn Biolabs, Foster City, CA, USA), 23 µL ultrapure water, 0.5 µL of each primer (concentration 33 µM), and 1 µL of DNA in DNAzol direct]. The PCR was performed using a thermal cycler TProfesional Basic (Biometra GmbH, Göttingen, Germany). Aliquots of the PCR products of volume 8 µL were mixed and stained with GelRed™ (Biotium Inc., Hayward, CA, USA). Stained PCR products were separated by horizontal 0.7% agarose gel electrophoresis in TAE buffer (pH 7.8) and visualized under UV light. The product of amplification at a volume of 15 µL was submitted for purification and sequence analysis to Microsynth (Microsynth AG, Wolfurt, Austria). The products were sequenced in both directions using either 27F or 1492R primer. Identification of selected bacterial colonies was based on BLAST analysis of sequences obtained after PCR for the 16S rRNA gene. Pairs of universal primers, 27F (5-AGAGTTTGATCMTGGCTCAG-3) and 1492R (5-CGGYTACCTTGTTACGACTT-3), were used for the PCR. The results were compared by BLAST analysis with the sequences available in the GenBank database.

### 2.12. Detection of Bacterial Microbiota Composition Based on NGS Amplicon Sequencing

Fecal samples from antibiotic-treated mice were used as a source for total DNA extraction. Total DNA was extracted from fecal samples using a ZR Fecal DNA MiniPrep™ commercial kit (Zymo Research, Irvine, CA, USA) according to manufacturer’s instructions. The 16S rRNA gene library was prepared using universal primers targeting the V3–V4 region (460 bp) [21]. PCR amplifications were performed in triplicate, were pooled and the gel was purified using a kit Wizard SV Gel and PCR Clean-Up System (Promega, Madison, WI, USA). The sequencing was performed using an Illumina MiSeq platform (2 × 250 bp) at the Genomics Core Facility (Universitat Pompeu Fabra, Barcelona, Spain). Initial processing of the obtained sequences was carried out in SEED2 [22]: reads were joined by means of the fastq-join function with default settings, the primers were cut off and the sequences were filtered for mean sequence quality > 30 and the correct length of the amplicon (approx. 420 bp). Chimera check was performed using UPARSE (built in SEED2) [23]. Further processing was carried out using Silva NGS online platform (https://www.arb-silva.de/ngs/), with OTU clustering threshold set at 98% similarity and other settings left at default. The raw unpaired sequence reads were submitted to the NCBI database under the BioProject identification number PRJNA636006.

### 2.13. Histological and Immunohistochemistry Analysis

The distal part of the colon (n = 6) was excised (both for histology and immunohistochemistry assessment), intensively washed in PBS (pH 7.2), and fixed in 4% paraformaldehyde in PBS for 72 h at 8 °C. After rinsing with water, the samples were dehydrated through a series of ethanol concentrations (100%, 96%, 90%, 70%) embedded in paraffin blocks and subjected to the classical procedure. Paraffin sections (7 µm thick) were deparaffinized, rehydrated in a graded alcohol series, and a part of histological sections was stained with Harrison’s hematoxylin and eosin (H&E) to determine the intensity of the inflammation and tissue damage. Other sections were stained with 0.9% Alcian Blue solution followed by 0.045% Safranin solution, which enabled visualization of goblet cells and their secretions were seen as blue color. The stained sections were dehydrated, cleared and mounted in a Histochoice mounting medium (Amresco LLC, Solon, OH, USA). The HAI was determined on five non-overlapping sections per each colon/mouse and each group. Morphometric analysis was performed on the length of 50 villi and depth of 50 crypts using an Olympus Microscope BX51 and a Digital Analysis Imaging system “Analysis Docu” (Soft Imaging System 3.0, Prague, Czech Republic).

Paraffin sections were used for immunohistochemical localization of PCNA and anti-apoptotic Bcl-xL antigens (B-cell lymphoma-extra large). Antigen retrieval was performed by boiling the slides in 10 mM citrate buffer (pH 6.0) for 2 min followed by washing. To block the endogenous peroxidase activity, the slides were incubated in TBS (0.05 M Tris–HCl, 0.15 M NaCl, pH 7.6) with 0.3% H_2_O_2_ for 20 min and the nonspecific binding was blocked during incubation of sections in 1% bovine serum albumin (BSA) in TBS for 1 h at room temperature. The sections were then incubated with primary antibodies anti-PCNA (mouse monoclonal) or anti-Bcl-xL (mouse monoclonal) (Santa Cruz Biotechnology Inc., Dallas, TX, USA) both at a dilution of 1:250 in PBS overnight at 4 °C. After rinsing with TBST (TBS containing 0.1% Tween 20), the sections were incubated with secondary goat anti-mouse IgG antibodies (Dako REAL™ EnVision™/HRP, Rabbit/Mouse (ENV), ready-to-use, Dako, Denmark) for 2 h at room temperature. The sections were then rinsed in TBST followed by TBS and then incubated with diaminobenzidine (DAB) as a chromogen (Dako REAL™ DAB+ Chromogen, Dako, Denmark) resulting in development of the color reaction. The stained sections were rinsed with distilled water, counterstained with hematoxylin to visualize nuclei, dehydrated, and immersed in DPX (Distyrene Plasticiser and Xylene, Buchs, Switzerland). For negative control staining, incubation with the primary antibody was omitted. Photographic documentation was obtained using an optical microscope (Olympus BX43, Olympus Corporation, Tokyo, Japan) coupled to a camera (Olympus UC30, Olympus Corporation, Tokyo, Japan) and computer. To evaluate the intensity of the immunohistochemical reaction quantitatively, approximately six images from sections of each examined animal (n = 6 for each group) were analyzed by using a public-domain ImageJ software (National Institutes of Health, Bethesda, MD, USA). The outlines of all cells which demonstrated an immunopositive signal in the colon were marked manually and then the grey level (GL) of the marked areas was measured. The intensity of the IHC reaction was expressed as the relative optical density (ROD) of the DAB-brown reaction products and was calculated using the formula described by [24], where GL is the grey level of the stained area (specimen) and unstained area (background) and blank is the GL measured after the slide was removed from the light path.

$$ROD=\;\frac{\mathrm{ODspecimen}}{\mathrm{ODbackground}}=\;\frac{\log\;\left(\frac{GL_{blank}}{GL_{specimen}}\right)}{\log\left(\frac{GL_{blank}}{GL_{background}}\right)}$$

### 2.14. Evaluation of Histopathological Findings

Histological analyses and mucosal integrity were assessed and classified independently by two pathologists. Morphometric analysis was performed on at least 50 villi in 50 microscopic fields at 200× magnification for each experimental group of mice. The histopathology of the tissue defined as HAI was characterized by the presence of infiltration of inflammatory cells, crypt loss, goblet cell reduction, and epithelial erosions (Table 2). Goblet cell loss is a modified assessment according to the study by [25], that expresses the proportion (%) of the area stained with Alcian Blue in the experimental group of mice (AM5) compared to the control (C).

### 2.15. Statistical Analysis

Statistical analysis was performed using Statistic software GraphPad Prism 5.0 for Windows (GraphPad Software, San Diego, CA, USA). The data were evaluated statistically by one-way analysis of variance (ANOVA), followed by a multiple comparison Tukey’s test. Significant differences between the two groups of mice were tested using analysis of variance and unpaired Student’s *t*-test. The results are expressed as means ± SD. Differences were considered significant at *p* < 0.05. Correlations between the different variables of the mice were performed using the Pearson’s correlation test based on 95% confidence interval. *p* value < 0.05 was considered statistically significant.

## 3. Results

### 3.1. Viability of Cecal Microbiota after Administration of Antibiotics

Oral administration of amoxicillin in combination with clavulanic acid and *s.c.* administration of ciprofloxacin negatively affected viability of cecal microbiota of mice (Figure 1b–d), as determined by Fluorescence-Activated Cell Sorting (FACS).

After 5-day administration of ATB, the viability of bacteria (Figure 1c; 16.2 ± 1.6%) was significantly lower (*p* < 0.001) in comparison with viability before the ATB treatment (Figure 1b; 74.5 ± 2.14%). Viability of microorganisms in the cecum of animals that were kept after the initial 5 days in microbiologically controlled environment of gnotobiotic isolators was significantly higher (*p* < 0.001) after 10 days following the termination of the ATB treatment and reached the level of 54.9 ± 6.8% (Figure 1d). The viability of microorganisms was visualized by the viability fluorescent quick test VFQTOPF (Figure 1b–d) based on color difference between live (green) and dead (red) bacteria.

### 3.2. Detected Cultivable Bacteria

**Figure 2:**
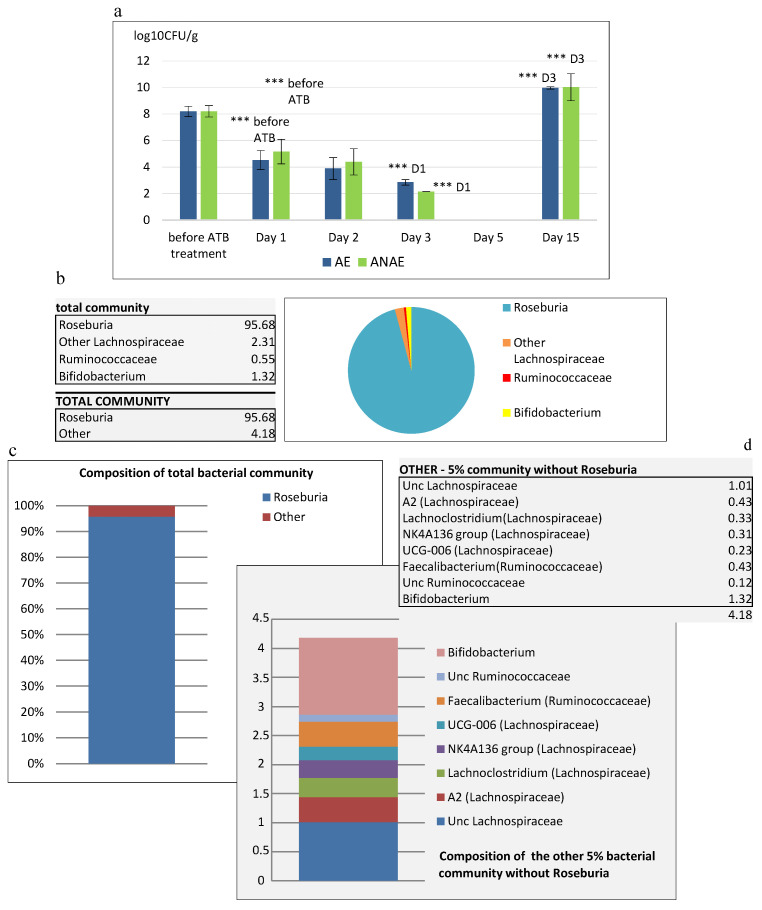
Microbiota composition in feces of antibiotic-treated mice. (**a**) Plate counts of microorganisms in feces determined by cultivation on TSA agar. AE aerobic conditions, ANAE anaerobic conditions. The results are expressed as the means log_10_ CFU/g ± SEM. *** *p* < 0.001. (**b**,**c**) Composition of total bacterial community. (**d**) Composition of the other 5% bacterial community without *Roseburia*. (**b**–**d**) Data are based on the sequences of the 16S rRNA gene (composite sample of 90).

Culture microbiological examination was carried out to observe in 24 h intervals the changes in the counts of cultivable bacteria caused by the ATB treatment. Before the treatment, the counts of cultivable bacteria in feces of all SPF mice were at the level of 8.2 log_10_ CFU/g (Figure 2a). After 24 h of the treatment with ATB, the counts decreased significantly (*p* < 0.001) by 4 log at aerobic cultivation (4.53 ± 0.14 log_10_ CFU/g) and by 3 log at anaerobic cultivation (5.17 ± 0.18 log_10_ CFU/g), compared to counts before the ATB treatment. On day 5 after administration of ATB, no cultivable bacteria were detected in samples of mice feces (Figure 2a). Significant recurrence of cultivable bacteria (*p* < 0.001) in mice feces was observed on day 10 after termination of ATB treatment when the counts at aerobic cultivation reached 9.75 ± 0.12 log_10_ CFU/g and those at anaerobic cultivation were 9.64 ± 0.13 log_10_ CFU/g.

Culture examination of feces, cecum, and gingival plaques of mice after 10-day convalescence allowed us to obtain two morphologically different types of colonies. BLAST analysis of the DNA sequence of the first isolate corresponding to 16S rRNA revealed maximum similarity with *Escherichia coli* (GenBank: CP 025910.1). For the second isolate, the BLAST analysis showed similarity with *Enterococcus galinarum* (GenBank: JN 412816.1).

### 3.3. Molecular Analysis of Gut Microbiota Composition after the Treatment with Antibiotics

We obtained 77,677 raw sequence reads. Upon data processing, filtering, and chimera check we lost approx. 15% of them and 65,158 sequences were assigned to taxonomy. We obtained 48 operational taxonomic units (OTUs) out of which 28 were singletons. Decrease in OTUs in conventional SPF mice to the level of several tens of OTUs, confirmed pronounced reduction in gut microbiota. We found that Clostridiales massively dominated the community and practically completely overgrew it (98.5%), with one major OTU identified as *Roseburia* (95%) (Figure 2b,c). Other smaller OTUs of Clostridiales (Figure 2d) were identified as A2 (0.4%), *Lachnoclostridium* (0.3%), NK4A136 group (0.3%) and UCG-006 (0.2%) and an uncultured group (1%) of Lachnospiraceae and *Faecalibacterium* (0.4%), and an uncultured group (0.1%) of Ruminococcaceae. Apart from Clostridiales, we also found 1.3% assigned to *Bifidobacterium* (Actinobacteria).

### 3.4. Production of SCFAs in the Cecum of Antibiotic-Treated Mice

The most pronounced decrease in production of organic acids (Table 3), particularly butyric (*p* < 0.001), acetic, and acetoacetic (*p* < 0.05) acids in the cecum of SPF mice (C5) was observed after 5-day administration of antibiotics which correlates with the absence of recovery of cultivable microorganisms from the feces of these animals and indicated a very low level of intestinal fermentation. The negative influence of administration of antibiotics on production of SCFAs was observed only for a short time period and already by day 10 following termination of their administration, the fermentation activity in the mice intestine (C15) was recovered and production of organic acids reached the level similar to that recorded before administration of ATB. The most pronounced significant increase was observed in butyric acid (*p* < 0.001) the level of which increased 5-fold in comparison with the situation after administration of ATB. In the post-convalesce period, concentrations of both acetic and acetoacetic acid in the investigated section of the digestive tract of SPF mice also reached significantly higher (*p* < 0.001) levels (Table 3). While the levels of these acids in C15 animals were relatively high and ranged from 89.00 to 134.0 mmol/L for acetoacetic acid and from 65.0 to 88.0 mmol/L for acetic acid, the concentrations of propionic, lactic, and succinic acids also increased significantly (*p* < 0.05) but their production did not suffice to exceed the level of 35 mmol/L.

### 3.5. Hematology Parameters

Significant differences in proportions of all observed parameters of the white blood component were observed particularly in groups AM3 and AM5 after 5-day exposure to DSS (Table 4) in comparison with the control group C. The highest total leukocyte counts (WBC) were observed in group AM5 (9.92 ± 3.12) where they reached 2.2-fold higher level in comparison with group C, and in group AM3 with significantly higher WBC counts (*p* < 0.01) compared to C. The groups AM3 and AM5 showed also higher counts of lymphocytes (Ly), monocytes (Mo), and granulocytes (Gran) in comparison with groups C and AM1. The counts of Ly and Gran in group AM3 were significantly higher (*p* < 0.01) in comparison with control group C.

Counts of parameters of the red blood component in groups AM3 and AM5 also differed significantly in comparison with control group C. In group AM5, that was exposed to 5% concentration of DSS, anemia due to acute hemorrhage was confirmed resulting in reduced counts of erythrocytes (RBC), decreased hematocrit (HCT), significantly higher (*p* < 0.05) concentration of hemoglobin (HGB), and higher mean corpuscular hemoglobin (MCH) in comparison with control group C.

Opposite trend was observed in the group exposed to 3% concentration of DSS (AM3). In this group we recorded significantly higher RBC counts (*p* < 0.05), higher but insignificant HCT, concentration of HGB, MCH, and mean corpuscular volume (MCV) in comparison with C that indicated relative polyglobulia caused by dehydration.

### 3.6. Evaluation of Severity of Clinical Colitis

**Figure 3:**
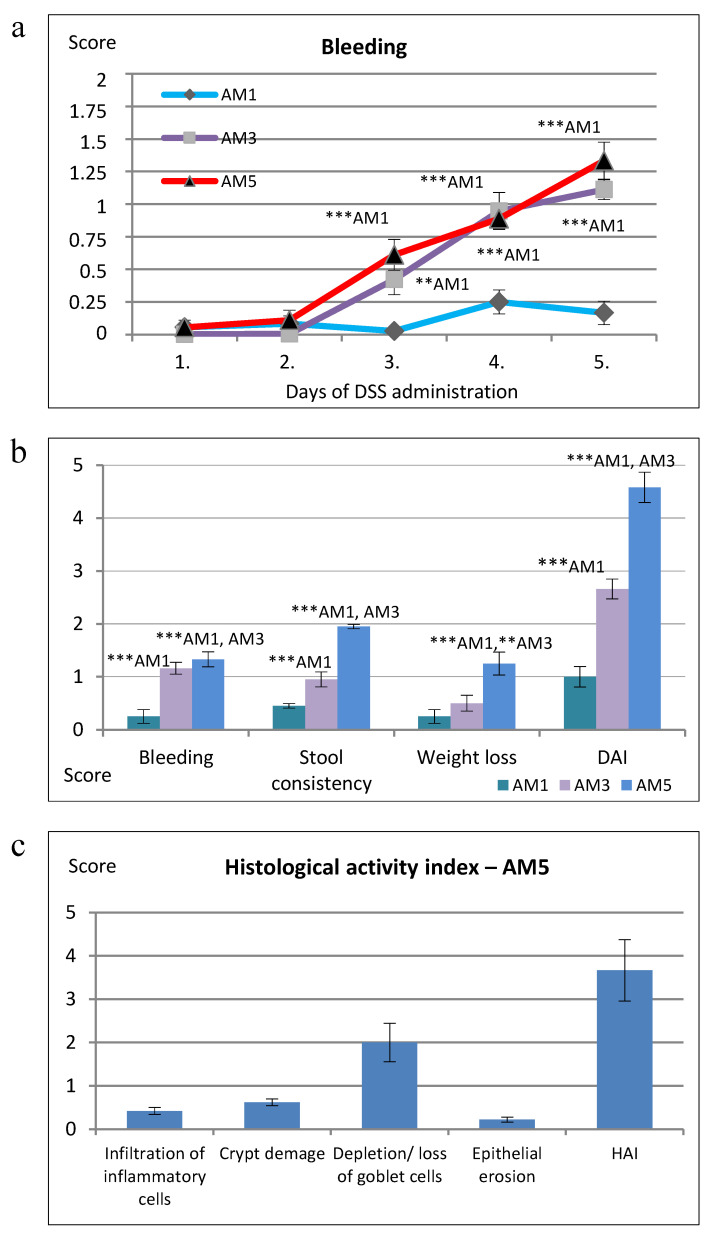
Disease Activity Index and histopathology score in mice with DSS-induced colitis. (**a**) Bleeding. (**b**) Clinical score after 5-day exposure to DSS. (**c**) Histological Activity Index in group AM5. AM1 (animal model 1% DSS, n = 18), AM3 (3% DSS, n = 18), AM5 (5% DSS, n = 18). DSS dextran sodium sulphate, DAI Disease Activity Index, HAI Histological Activity Index. The results are expressed as means ± SD. ** *p* < 0.01, *** *p* < 0.001.

Clinical evaluation of the inflammation induced by DSS included daily observation of body weight and overall health state of mice. The score of rectal bleeding (Figure 3a) recorded in groups exposed to 3% and 5% concentration of DSS (AM3, AM5) increased gradually from day 1 to 5 according to the severity of bleeding with the highest significance (*p* < 0.001) recorded on days 4 and 5 of DSS administration compared to group AM1. The group exposed for 5 days to 5% concentration of DSS (AM5), showed the highest total weight loss (*p* < 0.001, *p* < 0.01) in comparison with groups AM1 and AM3, respectively, that varied between 5% and 14% in individual animals (Figure 3b). The AM5 group exhibited also the highest score of rectal bleeding and differences in consistency of feces (*p* < 0.001), in comparison with groups (AM1 and AM3). Disease Activity Index (Figure 3b) increased gradually in this group and after 5-day exposure to DSS it reached the 4–6 levels of the 10-point scale in individual animals.

### 3.7. Evaluation of Histopathological Findings

The pathophysiology of the tissue defined as HAI was characterized by infiltration of inflammatory cells, crypt loss, goblet cell reduction, and epithelial erosion. In group AM5 the observed parameters reached the following HAI scores (Figure 3c): infiltration of inflammatory cells 0.42 ± 0.081; crypt damage 0.62 ± 0.08; goblet cell reduction 2.00 ± 0.443; epithelial erosion 0.22 ± 0.051; total HAI score 3.66 ± 0.70. In the group exposed to the highest concentration of DSS (AM5) we detected a significant positive correlation between DAI and the epithelial erosion (r = 0.864; *p* < 0.001) as well as between weight loss score and epithelial erosion (r = 0.734; *p* < 0.01). We recorded also a significant medium positive correlation between rectal bleeding score and epithelial erosion (r = 0.625; *p* < 0.05), attributed to a significant influence of erosive epithelium damage on increased bleeding, dehydration of an organism, and subsequent loss of total weight of mice. Within the histopathological score a significant, highly positive correlation was detected between total HAI score and goblet cell reduction (r = 0.912; *p* < 0.0001) and between infiltration of inflammatory cells and epithelial erosion (r = 0.864; *p* < 0.001).

### 3.8. Light Microscopy Results

**Figure 4:**
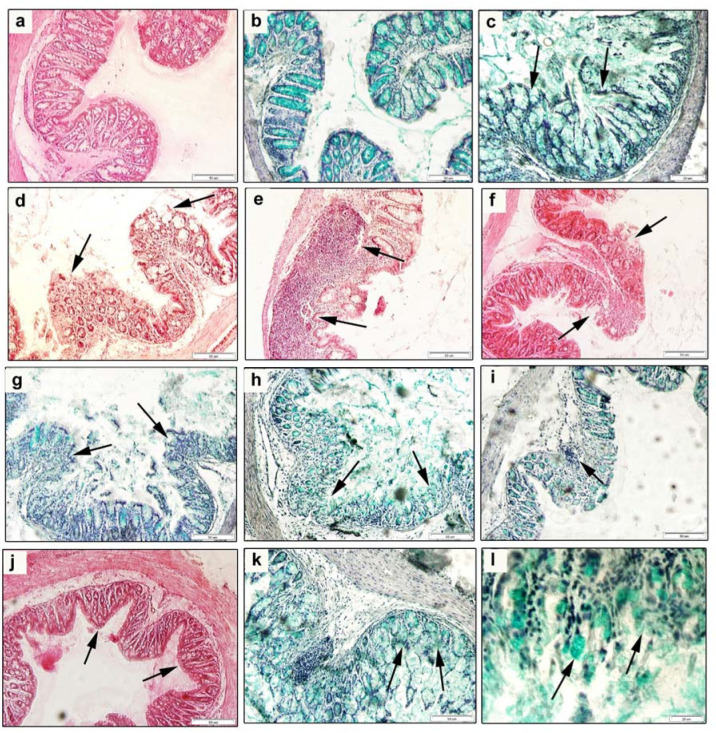
Histoarchitectural changes in the colon tissue after exposure to 5% DSS illustrating induction of acute colitis. (**a**,**b**) Healthy colon tissue in control animals (C) (H&E and Alcian blue; 100×). (**c**) Mucous defects with crypt fragments in the longitudinal section (Alcian blue; 200×). (**d**) Cross-section of adjacent mucus containing dilated crypts with variable diameters (H&E; 100×). (**e**–**i**) Leukocytic inflammatory infiltrate in *lamina propria* and *tunica submucosa* (H&E; Alcian blue; 100×). (**f**) Loss of epithelial cells (H&E; 100×). (**j**) Atrophy of villi (H&E; 100×). (**g**–**k**) Loss of cytoplasmic mucin related to loss of goblet cells (Alcian blue; 100×, 400×). (**h**–**l**) Presence of leukocytes in glandular lumen of intestinal crypts (cryptitis), preceding devastation of intestinal crypts (Alcian blue; 100×, 400×).

In comparison with the histological picture of the colon of healthy mice, examination of histological sections of the colon of experimental mice from group AM5 exposed to 5% DSS revealed considerable impairment of histological architecture of *tunica mucosa* and *tela submucosa* (Figure 4c–l).

*Tunica mucosa* and *tela submucosa* showed signs of infiltration with inflammatory cells of the leukocytic type (Figure 4e,i). Inflammatory infiltrates were observed even at the absence of defects in the epithelial barrier (Figure 4e). Surface erosions of *tunica mucosa* involved also its deeper layers including *lamina epithelialis mucosae* and *lamina propria mucosae*, and represented the source of rectal bleeding that we were able to detect (Figure 4f). The loss of surface cells of *tunica mucosa* had a direct negative effect on the number of goblet cells localized in its *lamina epithelialis mucosae* layer (Figure 4g,k). Smaller proportion of the area with affinity to Alcian blue resulted in decreased production of mucin in the colon lumen. We detected impairment of intestinal crypts in both cross and longitudinal histological sections. The following defects of crypts were observed: dilated crypts with variable diameters, cryptitis (Figure 4h) and crypt distortion, or their complete absence in the mucosa (Figure 4c,d). Impairment of the regular arrangement of intestinal crypts was related to the depth of erosion of the surface epithelial layer. Pathology of the histological architecture of *tunica mucosa* and *tela submucosa* of the colon that was observed in our study corresponded to the clinical picture of the acute ulcerative colitis.

### 3.9. Morphometric Parameters of the Colon

Light microscopy observation of the size of cross-sections of colon villi in the animals (Table 5) showed a significant reduction of cross-section of villi (*p* < 0.001) already in groups exposed to 1% and 3% concentrations of DSS in comparison with control animals (C). However, after 5 days of DSS administration, the cross-section of colon villi in mice exposed to 5% DSS (AM5) was significantly lower in comparison with control mice (C; *p* < 0.001) as well as with mice exposed to 1% and 3% DSS (*p* < 0.001 and *p* < 0.01, respectively). At the same time, we observed that in the same section of the intestine of AM5 mice the perimeter of villi was the lowest (811.8 µm ± 32.2 µm) and differed significantly by 268–353 µm from all other investigated groups. Similar negative influence of exposure to 5% DSS in group AM5 was observed with respect to the height of villi which was significantly reduced (*p* < 0.001) in comparison with the remaining groups (C, AM1, AM3). Continuous 5-day administration of DSS to animals from the AM5 group had an evident negative influence on all investigated morphometric parameters as this group exhibited also significantly lowest depth of crypts (*p* < 0.001) compared to groups C, AM1, and AM3 and the corresponding significantly highest villus height/crypt depth ratio in comparison with control group C (*p* < 0.001).

The presented study detected a negative influence of exposure to DSS on selected morphometric parameters also at lower DSS exposure, particularly the 3% concentration, that was manifested by significantly lower cross-section of villi, lower height of villi, and lower depth of crypts (*p* < 0.001).

### 3.10. Immunochemistry

**Figure 5:**
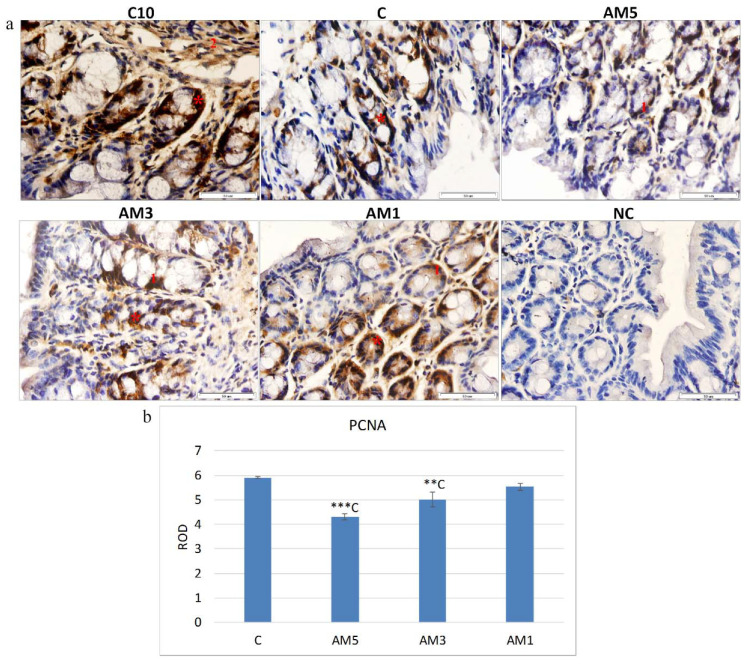
Immunohistochemical detection of PCNA in the intestinal wall of mice colon. (**a**) * Positive reaction at the crypt base and in the *lamina propria* layer: 1—positive reaction in the gland cells; 2—positive reaction in the *lamina propria* layer, NC image of the negative control (without primary antibody), scale bar 10 μm for PCNA, magnification 400×. (**b**) Quantification of the intensity of immunohistochemical reaction and expression of PCNA in mice colon, expressed as the relative optical density of the section (ROD). C10 (10 days without ATB), C (control, no DSS), AM1 (animal model 1% DSS), AM3 (3% DSS), AM5 (5% DSS). PCNA proliferating cell nuclear antigen. The results are expressed as means ± SD. ** *p* < 0.01, *** *p* < 0.001.

The influence of exposure to DSS in the colon tissue of mice was observed also with respect to the marker of cell proliferation (PCNA) which was detected by immunohistochemistry. Figure 5a shows immunohistochemical staining of the mice colon sections with PCNA-positive cells situated particularly at the basis of crypts and in *lamina propria*.

Quantification of the intensity of immunohistochemical reaction and PCNA expression in the same sections (Figure 5b), expressed as relative optical density (ROD) of the section, showed a significantly reduced cellular proliferation (*p <* 0.001, *p <* 0.01) in groups AM5 and AM3 in comparison with control group C. On the contrary, with decreasing concentration of DSS (group AM1) the cell proliferation increased. The negative effect of the exposure to 5% DSS on cell proliferation was confirmed by the observed medium negative correlation of the PCNA marker and HAI (r = −0.563) which indicated a relationship between increasing histological score and decreasing cellular proliferation. We recorded also a significant medium negative correlation between the goblet cell reduction score and the PCNA marker (r = −0.630; *p* < 0.05).

**Figure 6:**
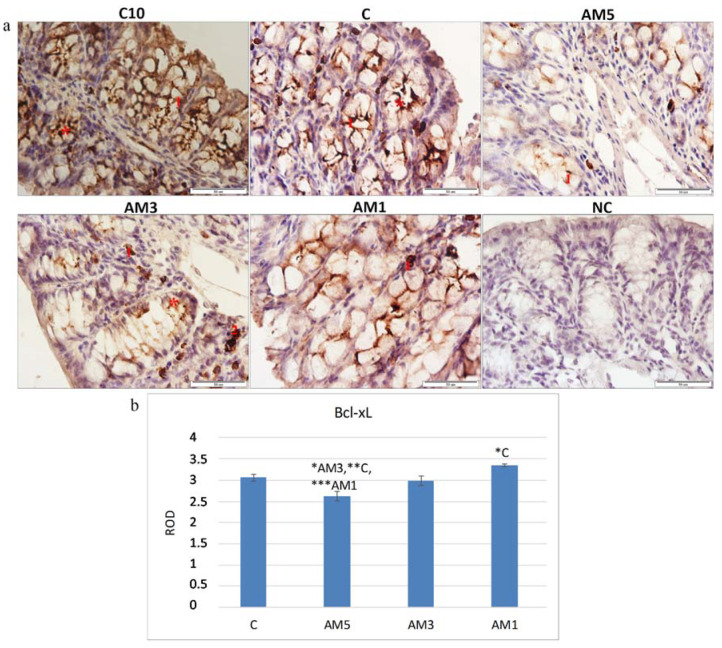
Immunohistochemical detection of the anti-apoptotic (Bcl-xL) marker in the intestinal wall of mice colon. (**a**) * Positive reaction at the crypt base and in the *lamina propria* layer: 1—positive reaction in the gland cells; 2—positive reaction in the *lamina propria* layer, NC image of negative control (without primary antibody), scale bar 50 μm for Bcl-xL, magnification 400×. (**b**) Quantification of the intensity of immunochemical reaction and expression of Bcl-xL in the mouse colon, expressed as a relative optical density of the section (ROD). C10 (10 days without ATB), C (control, without exposure to DSS), AM1 (animal model 1% DSS), AM3 (3% DSS), AM5 (5% DSS). The results are expressed as means ± SD. * *p* < 0.05, ** *p* < 0.01, *** *p* < 0.001.

Immunohistochemical analysis of the mice tissue colon was performed to detect the marker of cell apoptosis Bcl-xL. The Bcl-xL protein is a member of the Bcl-2 family of proteins within which it plays the role of an antiapoptotic marker, i.e., prevents leaking of cytochrome c from mitochondria and hinders apoptosis. Figure 6a shows immunohistochemical staining on the sections of mice colon. The Bcl-xL positive cells were situated mainly at the basis of crypts and in the *lamina propria* layer. The intensity of reaction decreased in the direction toward the surface—to epithelial cells. Quantification of Bcl-xL expression in the section of mice colon (Figure 6b), expressed as relative optical density (ROD) of the section, revealed a significant decrease in the presence of Bcl-xL marker (*p <* 0.05, *p <* 0.01, *p <* 0.001) in group AM5 in comparison with the remaining groups (AM3, C, and AM1). The negative influence of exposure to 5% DSS is confirmed by low negative correlation between the Bcl-xL and HAI score (r = −0.309), as well as low negative correlation between goblet cell reduction score and Bcl-xL (r = −0.337). On the contrary, we recorded a positive correlation between the marker of cell proliferation (PCNA) and Bcl-xL, although this correlation was low (r = 0.303). With the decreasing concentration of DSS the level of antiapoptotic marker increased as increased also the marker of cell proliferation PCNA. In the group exposed to the lowest concentration of DSS (AM1) we detected a positive stimulation effect on the level of the Bcl-xL manifested by its significant increase (*p <* 0.05) in comparison with control group C.

### 3.11. Viability of the Cecal Microbiota of PGF Mice Following the Exposure to DSS

**Figure 7:**
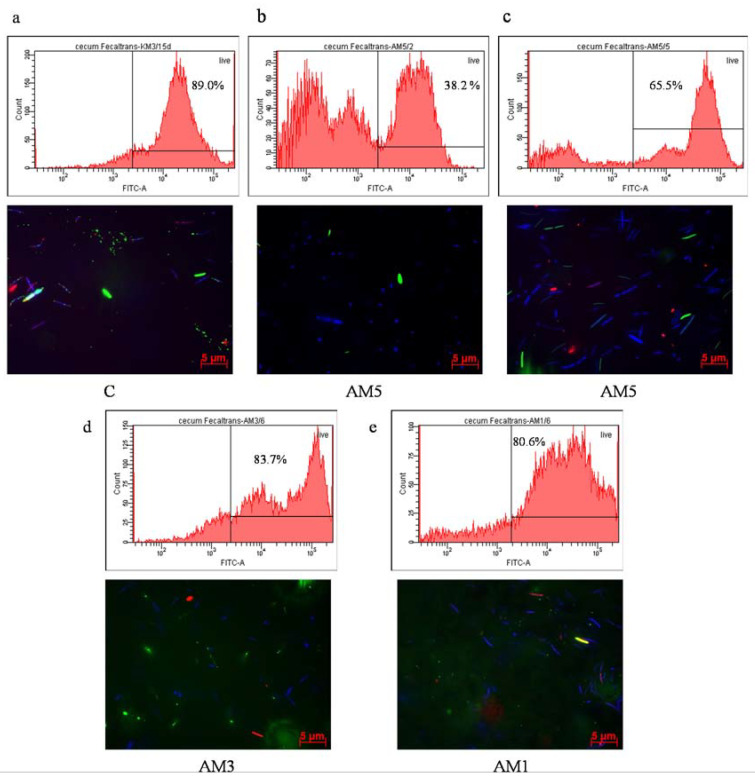
Viability of microorganisms in the cecum of mice following 5-day exposure to DSS determined by FACS (histograms showing percentage of live bacteria based on intensity of green fluorescence (X axis) opposite to counts (Y axis); living bacteria emit high intensity of green fluorescence) and visualized with VFQTOPF (VFQTOPF—viability fluorescent quick test on a polycarbonate filter; live bacteria are green, dead are red, and barely active but not dead are blue). (**a**) Viability of microbiota in the control group C not exposed to DSS. FACS analysis (89.0%) and VFQTOPF visualization. (**b**) Cecal content of mice exposed to 5% DSS (AM5), with high DAI score at the level of 5–6. FACS analysis (38.2%) and VFQTOPF visualization. (**c**) Cecal content of mice exposed to 5% DSS (AM5), with low DAI score at the level of 1–2. FACS analysis (65.5%) and VFQTOPF visualization. (**d**) Viability of microbiota in mice from group AM3 exposed to 3% DSS. FACS analysis (83.7%) and VFQTOPF visualization. (**e**) Viability of microbiota in mice from group AM1 exposed to 1% DSS. FACS analysis (80.6%) and VFQTOPF visualization. DAI—disease activity index score.

Exposure to dextran sodium sulphate resulted in decreased viability of cecal microbiota (Figure 7) compared to that in the control group C (86.70%). The decrease was significant (61.42%; *p* < 0.01) in group AM5 exposed to 5% concentration of DSS. Noteworthy is the dependence on the intensity of DAI, as at its low intensity with score 1–2 the viability of cecal microbiota in this group reached the level of 74.4%, while the high intensity DAI with inflammation score of 5–6 resulted in the viability of microbiota at the level of 38.2%.

While the exposure of mice from group AM5 to 5% DSS had a negative influence on the viability of microorganisms in the cecal content, exposure to 3% and 1% concentrations of DSS failed to produce a marked negative influence on the viability of cecal microbiota in groups AM3 and AM1, where it varied at 84.7 ± 0.55% and 73.57 ± 3.5%, respectively. Figure 7 shows the viability of microbiota in the cecum of mice visualized by the method VFQTOPF, based on color detection of live bacteria (green) and red bacteria (red).

## 4. Discussion

All kinds of animal models can presently be used to study the processes involved in intestinal inflammation. Animal models of IBD have been a part of preclinical research for more than five decades. However, rodent models and particularly the genetically modified inbred mice strains with highly homogeneous genetic makeup that increases reproducibility of results and the strength of statistical evidence of experiments are the primary models that have been used in the studies of acute and chronic inflammation of the gut. In addition to selection of the best animal model used for examination of specific aspects of intestinal inflammation, one must consider carefully which chemical compounds and biological agents are most effective in inducing the inflammation [12]. None of the models can embrace the complexity of human IBD, but every model provides valuable knowledge and all of them together can create a generally accepted set of principles of human IBD pathogenesis [28]. The humanized gnotobiotic animal model that comprises an accurately identified complex of microorganisms plays an important role in the studies of various pathogeneses. However, Chirlaque et al. [18] concluded that the intestinal bacteria are inevitable for the development of common DSS-induced colitis. Absence of microbiota significantly decreases inflammation of the large intestine after exposure to DSS, but also impairs the function of the intestinal barrier. The importance of the presence of remnant microbiota for the development of model colitis was confirmed by the studies of a number of authors based on antibiotic treatment or pseudo germ-free (PGF) animals [29,30,31]. Therefore, our procedures were carried out on animals that developed physiologically under conventional conditions but were kept temporarily at the absence of microorganisms in their environment. Sterilization of the intestine by administration of antibiotics *per os* or *s.c.* facilitates the study of physiology of nutritionally important relationship between gut microbiota and the host.

In the 1st phase of our study, we decontaminated the digestive tract of SPF mice of BALB/c line with antibiotics according the described procedures [19,20]. The antibiotics were selected on the basis of culture methods as recommended by Johnson et al. [32], in order to eliminate ATB with a serious negative effect on the health of animals. The mice were decontaminated by oral gavage feeding of amoxicillin at a dose of 387.11 mg/kg/mouse and subcutaneously administered ciprofloxacin at a dose of 19.60 mg/kg/mouse while keeping the animals in exactly defined environment of gnotobiotic isolators. Similar to our previous study, flow cytometry was used [19] to confirm the negative influence of 5-day antibiotic decontamination on the viability of cecal microbiota of mice. We recorded a significant decrease in activity of gut microbiota to the level of 16.2% (*p* < 0.001) in comparison with the situation before administration of ATB (74.5%). A significant increase in the viability of cecal microorganisms (*p* < 0.001) was observed following the 10-day period after termination of administration of ATB which reached the level of 54.9%. A two-week or even shorter administration of broad-spectrum antibiotics may decrease the bacterial counts by several orders of magnitude [33,34]. Though a broad-spectrum antibiotic approach considerably reduces the majority of the bacterial species a certain portion of microorganisms persists in the intestine, as was determined by culture or molecular methods with some delay after antibiotic decontamination [35]. This was confirmed also by our study, as before administration of ATB the counts of cultivable microorganisms in the feces of SPF mice reached the level of 8.2 log_10_ CFU/g, no cultivable microorganisms were detected in the feces after 5-day administration of ATB, but after 10 days following the termination of ATB treatment they were again present at a significant level (*p* < 0.001). With regard to the differences in the mechanisms of their action, different ATB may selectively inhibit different members of gut microbiota. For example, vancomycin is effective only against Gram-positive bacteria, ciprofloxacin reduces aerobic bacteria [36], metronidazole and clindamycin act on anaerobes while polymyxin B selectively inhibits Gram-negative bacteria [31,37,38]. Administration of various antibiotic cocktails based on nondefined individual doses of the individual components per animal presents the risk of the absence of control of the exact effect of the antibiotic treatment with respect to the complete eradication of any bacterial species or only inhibition of their growth. It is exactly the persisting microbiota of the mice treated with antibiotics that can have a decisive influence on colonization of the intestine and can result in increased counts of some species, for example *Klebsiella* spp., with considerable negative influence on the animal health [35], not considering the fact that this represents an uncontrolled situation with negative influence on reproducibility of studies. The methodical procedures regarding administration of antibiotics used in this study were the same as those used in the previous one [19]. Cultivation of samples of feces and the content of cecum confirmed the presence of two types of morphologically different colonies. BLAST analysis of the DNA sequence that corresponded to 16S rRNA revealed the highest similarity with the species *Escherichia coli* (GenBank: CP 025910.1) and *Enterococcus gallinarum* (GenBank: JN 412816.1) which corresponded to our previous results. Successful repeatability of the identification of bacterial sequences was confirmed at the level of species but with different accession number of the GenBank database. Our results obtained by analysis of samples of mice feces and their cecal content corresponded to those obtained by analysis of gingival plaque samples, which was an expected finding due to coprophagy of rodents. Sun et al. [39] observed the ability of vancomycin, enrofloxacin, and polymyxin B to modify the intestinal microbial community and metabolism. These authors observed that before administration of ATB, Bacteroidetes and Firmicutes were the most predominant phyla in mice. Administration of vancomycin for the period of 3 weeks induced significant changes in bacterial composition and richness. These changes were reflected in a significant increase particularly in bacteria belonging to the Proteobacteria phylum (from 1.7% to 52.1%) and, on the contrary, reduction of bacteria belonging to the phyla Bacteroidetes (from 59.3% to 5.5%) and Firmicutes (from 37.1% to 10.4%). Similar abundance of bacteria at the level of phylum in favor of Proteobacteria and a significant decrease in Bacteroidetes and Firmicutes were observed by a number of authors [40,41,42], who administered for more than two weeks an antibiotic cocktail that comprised four ATB (ampicillin, vancomycin, metronidazole, neomycin) and one antifungal drug (amphotericin B) [43]. Our study showed that the 5-day individual administration of the amoxicilin and ciprofloxacin combination caused a significant reduction in composition of intestinal microbiota in favor of phylum Firmicutes (98.54%). When the analysis was performed at lower taxonomic ranks by Sun et al. [39], the authors observed more pronounced changes in some other taxa. The investigation of 3-week administration of vancomycin showed an increase in bacterial abundance at family level, particularly of Enterobacteriaceae from 0.6% to 45.7% and of Akkermansiaceae from 0.7% to 19.1%. On the contrary, a decrease in abundance of Muribaculaceae from 43.8% to 3.5%, Lachnospiraceae from 25.3% to 3.6%, and a less pronounced decrease in abundance of families Prevotellaceae, Ruminococcaceae, and Bacteroidaceae was observed. The decrease in abundance of OTUs (several thousands) to the level of several tens of OTUs, observed in conventional SPF mice in our study, indicated a significant reduction in intestinal microbiota. We found that the order Clostridiales massively dominated the community and practically completely overgrew it (98.5%), with one major OTU identified as *Roseburia* (95%).

Contrary to *Lactobacillus* and *Bifidobacterium*, *Roseburia* is one of the less abundant bacterial genera that nevertheless plays an important role in the intestinal ecosystem. In the healthy intestine it comes from 3–15% of the total bacterial count [44]. Many studies confirmed a direct relationship between *Roseburia* and gut health, particularly with respect to the pathogenesis of IBD and cancer of the large intestine [44,45,46]. A significant reduction in butyric acid producing bacteria, particularly *Roseburia* spp., was observed in patients with diagnosed UC or CD [45], and was also revealed that this is a specific characteristic of their intestinal microbiota [46]. At the same time, there was observed a positive correlation between lower abundance of *Roseburia* and lower production of SCFAs, particularly butyrate [44]. SCFAs are the main metabolite produced by intestinal bacteria during fiber fermentation and their abundance in the digestive tract reflects the level of intestinal fermentation. Fermentative substrates of some species serve as substrates for fermentation or are incorporated as intermediate metabolites into the metabolic pathways of other species which results in gradual fermentation of substrates [47]. The main final products of sugar catabolism are SCFAs, such as acetate, propionate, and butyrate, the production of which is responsible for their 85–95% abundance in the total SCFAs in the colon section. Other final products of fermentation, such as caproate and valerate, are present in smaller amounts [48].

SCFAs fulfil an important protective function against intestinal infection, hinder the development or absorption of toxic products of the metabolism, maintain integrity of mucosa, and support the growth of epithelial cells in the large intestine [49]. It has been assumed that the level of organic acids in the digestive tract of animals that lack gut microbiota, such as germ-free or gnotobiotic animals, is very low. Our previous investigations [50] confirmed very low fermentation activity in the feces of germ-free and gnotobiotic mice with *Bacillus licheniformis* monoculture. The concentration of all investigated acids in these animals did not exceed 12 mmol/L. Higher concentrations of SCFAs in conventional mice in comparison with GF animals were also recorded by [51,52]. Low fermentation level in the digestive tract was confirmed also in the CB6F1 line of SPF mice treated with two different combinations of broad-spectrum antibiotics comprising vancomycin, metronidazole, neomycin, ampicillin and gentamycin, ciprofloxacin, streptomycin, bacitracin, that significantly decreased concentrations of acetate and butyrate [53]. Low production of SCFAs, particularly lactic, acetic, propionic, succinic and butyric acids was detected also in our study in SPF BALB/c mice treated with antibiotics amoxicillin and cipofloxacin [20]. Yan et al. [53] observed that administration of vancomycin alone sufficed to decrease SCFAs to the same extent as a broad-spectrum antibiotic therapy. Metagenomic analysis significantly facilitated identification of bacterial species responsible for production of SCFAs.

The significant increase in SCFAs, particularly of production of butyric acid observed in our study 10 days following the termination of ATB treatment can be attributed to higher abundance of butyrate-producing anaerobes, such as *Roseburia* spp. Commensal microorganisms *Roseburia* spp. are producers of SCFAs metabolites, such as butyric acid [54,55,56,57] and propionic acid [55]. Our assumption is that in our antibiotic treatment model also representatives of *Faecalibacterium* spp. (0.43%) and *Ruminococcus* spp. (0.55%) participated to a minor degree in production of butyrate, as they also belong to producers of this metabolite [54,55,56,57,58]. Butyrate is an important energetic substrate in the colon that also stimulates the growth of epithelial cells [44,59]. Increased abundance of the representatives of the genus *Roseburia* and the concomitant increased production of butyrate after the period of antibiotic decontamination can represent an important factor of stabilization of the digestive tract ecosystem, as it can contribute to optimization of the digestive processes and increased resistance of mucosa to destruction. The parallel recovery of production of acetate and lactate may probably be attributed to 1.32% abundance of *Bifidobacterium* spp., the producer of both these metabolites [54], in partnership with acetate producing *Ruminococcus* spp. [54,55,57,58].

Convalescence of decontaminated animals under gnotobiotic conditions for the period of 10 days resulted in returning of hematologic, metabolic, and morphological parameters to the physiological range [20] while the species richness of mice microbiota was not restored. Results presented in this study as well as those obtained previously allowed us to obtain a pseudo germ-free model with reduced microbiota (the counts of cultivable microorganisms were reduced to two species, *Escherichia coli* and *Enterococcus gallinarum,* and the intestinal microbiota was reduced in favor of the genus *Roseburia*) without alteration of the total health state of the animals. This way of obtaining animals can be used in further studies focused on modulation of the intestinal microbiota.

In the 2nd stage of the experiment, the pseudo germ-free (PGF) model mice of BALB/c line were used to investigate pathogenesis of ulcerative colitis induced by different concentrations of DSS. DSS is a sulphate polysaccharide with highly variable molecular mass (Mr) ranging from 5 to 1400 kDa. It has been used extensively in research to induce intestinal colitis and cancer of the large intestine and rectum in mice and rats. The seriousness of DSS induced colitis [60] differs according to the molecular mass (i.e., 5, 40, and 500 kDa) of the administered DSS. The most serious colitis in BALB/c mice developed after administration of DSS of molecular mass of 40 kDa, while the laboratory animals exposed to DSS of molecular mass only 5 kDa developed only milder form of colitis. Similar results were obtained by Hirono et al. [61] after induction of carcinogenic activity in the colon of mice by DSS (54 kDa), while DSS of higher (520 kDa) or lower Mr (9.5 kDa) failed to induce any carcinogenic activity. The studies that investigated absorption and distribution of DSS in the tissues by means of histochemical methods proved that the failure of DSS of Mr equal to 500 kDa is caused by its high molecular mass that prevents the passage of DSS molecules through mucous membranes [60]. In our study, we used DSS of molecular mass of 40 kDa and administered it at 1%, 3%, and 5% concentrations.

Determination of the clinical activity of colitis involved the daily monitoring of score of stool consistency, blood in the stool, and weight loss during administration of DSS. On the basis of their earlier study (data not presented), the authors of [62] found out that the induction of UC in mice of C57BL/6 line with DSS of molecular mass 45 kDa at 5% concentration and 7-day exposure elicited marked symptoms of acute colitis resulting in the death of animals. Therefore, in their study published in 2005 [62] these authors selected DSS of the same mass but at lower concentration (3%) and 5-day administration. For comparison, the same authors used 5% concentration and the same length of exposure at BALB/c mice. Higher weight losses in C57BL/6 mice were recorded from day 4 of exposure to DSS, and in the study by Nunes et al. [63] from day 5. The weight losses by day 5 amounted to 5–8%, however, the gradual decrease of weight in the following observation period resulted in a total weight loss of almost 18% by days 8–10. Contrary to this, in experiments with BALB/c mice [62,64], despite higher exposure to DSS, the highest losses of weight were observed only from day 6 and the maximum losses reached 5–8%. Similar results were obtained in our study with more pronounced weight losses in BALB/c mice not detected before day 5 of exposure to DSS, and while the losses ranged from 5% to 14%, their mean level was only 8%. Experimental colitis induced by DSS has been associated with rectal bleeding and subclinical hematochezia in model animals [18]. Such bleeding is consistent with the mechanism of epithelial damage, particularly due to the action of DSS on subepithelial cells, which results in capillary lesions and loss of blood in the lumen. As the DSS is administered continuously, this process goes on without end and long-term bleeding ensues [18]. In the colitis model involving C57BL/6 mice the bleeding was more pronounced [62] and on day 5 reached the score of 1.7, while in the BALB/c line the score was significantly lower and did not exceed the level of 0.5. In our model of colitis, induced by 5% DSS, the score of rectal bleeding determined on day 5 in group AM5 reached 1.3. The rectal bleeding score increased gradually from day 1 to 5, according to the extent of inflammation, with significant difference (*p* < 0.001) on days 4 and 5 of exposure to DSS in comparison with AM1 (1% DSS). These results were confirmed by hematological findings in mice from group AM5 which supported the diagnosis of anemia due to acute hemorrhage. At the same time, there was detected a significant positive medium correlation between the score of bleeding and epithelial erosion (r = 0.625; *p* < 0.05) and high positive correlation between DAI and epithelial erosion (r = 0.864; *p* < 0.001) as well as the weight loss score and epithelial erosion (r = 0.734; *p* < 0.01). Erosion is defined as a loss of surface epithelium with underlying inflammation where the epithelial defect crossed the basal membrane and its manifestation is mostly focal [65].

Pathogenesis of ulcerative colitis is directly related to the infiltration of mucosa of the colon with leukocytes. It is well known that infiltration of neutrophils into the mucosa is one of the most important events of colon inflammation during the acute phase of the colitis. The histological sections of the colon of AM5 mice obtained in our study showed considerably impaired histological architecture of *tunica mucosa* and *tela submucosa,* that were affected by inflammatory infiltration with leukocytic cells. Within the histopathological score, we observed a significantly high positive correlation between infiltration of inflammatory cells and epithelial erosion (r = 0.864; *p* < 0.001). These findings correspond to the statements of the authors of studies in which DSS was used to induce colitis in mice [66,67,68]. Serious or long-lasting inflammation results in deformation of the architecture of crypts involving their irregular shape or even loss [25]. Our study showed deformation of intestinal crypts in the group AM5, manifested by cryptitis and crypt distortion or their complete loss at the level of score of 0.62 which indicated that the crypt damage involved only some of them. Our results agree with those of Perše and Cerar [69]. In their study, after exposure of mice of C57BL/6JOlaHsd line to DSS, half of the animals showed cryptitis or abscesses of crypts, but cryptitis was rare in the mice of BALB/cAnNHsd line subjected to DSS exposure. Determination of the selected morphometric parameters indicated also changes in mucosal architecture related to the appearance of villi in comparison with control animals. They involved lower cross-section of villi, their decreased height and lower depth of crypts (*p* < 0.001). The gradual widening and blunting of villi, progressing to complete loss of villous structure, i.e., their atrophy, indicated clearly involvement of pathological changes. Another accompanying histological characteristic of acute colitis induced by exposure to DSS is the depletion of mucus. At ulcerative colitis, the abundance as well as the size of goblet cells are reduced, and it is still not known whether the changes in production of mucin contribute to initiation of inflammation or result from this process [25]. As concerns the product secreted by goblet cells, it is characterized as depletion of mucin [70] or depletion of production of mucin by goblet cells [71]. The goblet cell loss in experimental group of mice (AM5) detected in our study compared to the control (C) was observed at the level of score equal to 2.00, i.e., maximum 35% loss, which corresponds to the mild depletion. Within the histopathological score we detected a significant very high positive correlation between the total HAI score vs. goblet cell reduction (r = 0.912; *p* < 0.0001). Ulcerative colitis impairs the balance between proliferation and apoptosis of epithelial cells [72]. The most commonly used markers of proliferation are PCNA and Ki-67. It is well known that the PCNA antigen participates not only in cellular proliferation but also in restoration of DNA after its damage [73]. In the presented study, the marker of cellular proliferation was detected in the section of mice colon, with PCNA positive cells situated particularly at the base of crypts and in *lamina propria,* which is in agreement with [74] who stated that PCNA immunohistochemistry can be used as a reliable marker of the proliferative compartment in both normal and neoplastic colonic mucosa of rats. Quantification of the intensity of immunohistochemical reaction and expression of PCNA in our study, indicated a significantly reduced cellular proliferation (*p <* 0.001) particularly in the group AM5 in comparison with control group C. On the contrary, with decreasing concentration of DSS (group AM1) the cell proliferation increased. The negative influence of exposure to 5% DSS on cellular proliferation was reflected also in the recorded negative medium correlation between the PCNA marker and HAI (r = −0.563), indicating a relationship between the increasing histological score and the decreasing cellular proliferation. These results agree with those of [75], who induced acute colitis in BALB/c mice by their exposure to 3% DSS and recorded changes in cellular processes characterized by a decrease in the marker of cellular proliferation (PCNA). The histochemical analysis of the colon tissue of mice identified also a marker of cellular apoptosis Bcl-xL. The Bcl-xL protein is a part of the Bcl-2 family of proteins within which it plays the role of an antiapoptotic marker. Recently, this protein has gained on importance as it serves as a marker of successfulness of IBD treatment. Adachi et al. [76] and Weder et al. [77], described in their studies increased expression of the Bcl-xL, as a marker of successful treatment of IBD and of restoration of the balance between proliferation and apoptosis of erythrocyte cells. A significant decrease in the Bcl-xL marker in group AM5 (*p <* 0.05, *p <* 0.01, and *p <* 0.001) was observed in our study in comparison with all remaining groups (AM3, C, and AM1, respectively). The negative influence of the exposure to 5% DSS was corroborated also by the low negative correlation between the Bcl-xL marker and HAI score (r = −0.309). On the contrary, we observed a positive although low correlation between the marker of cellular proliferation (PCNA) and Bcl-xL (r = 0.303). Contrariwise, with decreasing concentration of DSS the level of the antiapoptotic marker increased, same as the level of the marker of cellular proliferation PCNA. After exposure to 5% DSS the viability of cecal microbiota also decreased. A remarkable observation was its dependence on the intensity of DAI, as the viability of cecal microbiota in group AM5 reached 74.4% at low intensity of DAI (score 1–2) but only 38.2% at the score of 5–6.

Due to its low cost and relatively easy applicability, the DSS-induced model is one of the models most frequently used in the studies of various aspects of IBD, such as pathogenesis, genetic predisposition to IBD, immune mechanisms, and role of microbiota in the pathogenesis of IBD [69]. However, as indicated also by the results of this study, obtaining the optimum animal model of acute UC by means of exposure to DSS depends on many factors that may potentially affect the results, such as the susceptibility of the mice strain, selection of suitable administration scheme and antibiotic-treatment with respect to the residual gut microbiota, molecular mass of DSS and its concentration, and the length of exposure to DSS.

This study indicates that the changes induced by standardization of the procedure involving selective antibiotic decontamination of intestinal microbiota do not contribute to conditions supporting inflammation. This is confirmed by the presence of reduced microbiota and the fact that the family Clostridiales massively dominated the community and practically completely overgrew it (98.5%), with one major OTU identified as *Roseburia* (95%). The induced dysbiosis did not serve as a bearer of pathogenic microorganisms participating in development of ulcerative colitis. The composition of intestinal microbiota probably did not result in disturbance of mechanisms involved in resistance to colonization, growth, and production predominantly of *Clostridium difficile* and its toxins. These toxins impair epithelial cells and activate inflammatory responses that also support infection [78]. Recovery of butyrate and its adequate production in mice following their convalescence should create conditions for improved integrity of intestinal mucosa. In our study, the obtained PGF model did not exhibit changes that could contribute to inflammation by means of alteration of the metabolic status and the inflammatory process developed mostly due to the exposure to DSS and its toxic action on compactness and integrity of mucosal barrier in the large intestine. This offers new possibilities of the use of this animal model in studies with or without participation of pathogenic microbiota in IBD pathogenesis because, at present, it is still unclear whether the dysbiotic microbiota found in many patients with IBD really plays a causative role or, alternatively, it only reflects the inflammatory and antimicrobial responses developed during the disease.

There were some limitations to the study. Firstly, we should mention that detection of bacterial microbiota composition in mice after their antibiotic decontamination was based on NGS amplicon sequencing but the initial status was determined by identification of cultivable bacteria. Although the results were supported by the viability of microorganisms in the cecum of mice determined after simultaneous staining of samples with carboxyfluorescein diacetate and propidium iodide using flow cytometer and epifluorescence microscope, detection of bacterial microbiota composition based on NGS amplicon sequencing would provide more relevant results. Secondly, observation of relevant specific criteria of ulcerative colitis in our study involved evaluation of DAI, hematology parameters and HAI, supported by morphometric parameters, cell proliferation (PCNA) marker, Bcl-xL marker, and viability of microorganisms. For more precise interpretation of the inflammatory process, the above evaluations should be extended by additional important inflammation markers. The follow-up studies should include also observation of (iNOS) and cyclo-oxygenase 2 (COX-2). Thirdly, our study lacks information about the total and local immunological responses following the exposure to DSS, particularly observation of expression of proinflammatory cytokines (IFN-y, IL-10, and IL-12). Their observation as well as determination of subpopulations of lymphocytes CD4+, CD8+, CD4+CD8+, CD49b+CD8+, CD49b+CD8+, and CD4:CD8 ratio, will be the subject of another manuscript reporting results of a follow-up of this study.

## 5. Conclusions

By decontamination with selective antibiotics and observation of pathogenesis of ulcerative colitis induced chemically by exposure of mice to various concentrations of DSS, we obtained an optimum animal PGF model of acute UC manifested by mucin depletion, epithelial degeneration, and necrosis, leading to the disappearance of epithelial cells, infiltration of *lamina propria* and submucosa with neutrophils, cryptitis, and accompanied by decreased viability of intestinal microbiota, loss of body weight, dehydration, moderate rectal bleeding, and a decrease in the selected markers of cellular proliferation and apoptosis. The obtained PGF model did not exhibit changes that could contribute to inflammation by means of alteration of the metabolic status and the induced dysbiosis did not serve as a bearer of pathogenic microorganisms participating in development of ulcerative colitis. The inflammatory process was induced particularly by exposure to DSS and its toxic action on compactness and integrity of mucosal barrier in the large intestine. This offers new possibilities of the use of this animal model in studies with or without participation of pathogenic microbiota in IBD pathogenesis. The optimum induction of acute UC by exposure to 5% DSS will subsequently be used in procedures involving transplantation of fecal microbiota to the PGF animal model.

## Figures and Tables

**Table 1:** Disease Activity Index (DAI) score.

Score	Weight Loss	Stool Consistency	Bleeding	Maximum Score
0	No weight loss	Formed	No bleeding	10
1	5–10%	Mild soft	Few blood-tinged stools	
2	11–15%	Very soft	Slight bleeding	
3	16–20%	Watery stool	Gross bleeding	
4	>20%	-	-	

**Table 2:** Histological Activity Index (HAI) of the colon.

Grade (Score)	Infiltration of Inflammatory Cells	Crypt Damage	Depletion/Loss of Goblet Cells	Epithelial Erosion
0	Absence of infiltrate	None	None	Morphologically normal
1	Infiltrate at the subepithelial and *lamina propria*	Some crypt damage, spaces between crypts	Minimal (<20%)	Focal destruction
2	Infiltrate reaches *muscularis mucosae*	Large spaces between crypts	Mild (21–35%)	Zonal destruction
3	Severe and extensive infiltrate reaching submucosa and involving *muscularis propria*	Large spaces without crypts, surrounded by normal crypts	Moderate (36–50%)	Diffuse and mucosal ulcerations
4	-	No crypts	Marked (>50%)	-
References	[26]	[27]	[25]	[26]

The sum of the scores of four parameters was defined as the mucosal damage score, with maximum score 14.

**Table 3:** Concentration of SCFAs in the cecum of BALB/c mice before and after administration of ATB.

Group	Acid (mmol/L)						
	Lactic	Acetic	Succinic	Acetoacetic	Propionic	Butyric	Valeric
C0 (n = 126)	14.08 ± 3.24	86.65 ± 12.92	16.19 ± 4.83	65.87 ± 11.2	27.52 ± 7.86	33.04 ± 1.65	10.91 ± 2.87
C5 (n = 108)	9.83 ± 1.05	27.28 ± 5.20 * C0	18.5 ± 3.87	26.86 ± 3.24 * C0	17.58 ± 2.81	6.83 ± 1.18 *** C0	5.14 ± 1.66
C15 (n = 90)	14.15 ± 1.17 * C5	107.7 ± 6.31 *** C5	34.02 ± 4.28 * C5	78.86 ± 3.40 *** C5	26.09 ± 1.64 * C5	59.24 ± 9.99 *** C5	9.18 ± 1.85

C0 concentration in the mice cecum before antibiotic treatment. C5 concentration in the mice cecum on day 5 after ATB treatment. C15 concentration in the mice cecum after 10 days without ATB treatment. Results are presented as means ± SD. * p < 0.05, *** p < 0.001.

**Table 4:** Hematology parameters of the pseudo GF (PGF) mice following 5-day exposure to DSS.

Group	C	AM1	AM3	AM5	Ref BALB/c
WBC (G/L)	4.40 ± 0.55	4.77 ± 0.55	8.28 ± 0.77 ** C	9.92 ± 3.12	5.69–9.87
Ly (G/L)	3.45 ± 0.41	3.57 ± 0.27	5.30 ± 0.24 ** C *** AM1	6.05 ± 1.76	3.60–7.29
Mo (G/L)	0.15 ± 0.03	0.15 ± 0.02	0.51 ± 0.17	0.38 ± 0.14	0.34–0.70
Gran (G/L)	0.78 ± 0.11	1.04 ± 0.15	2.42 ± 0.56 ** C	2.26 ± 0.96	0.74–1.78
RBC (T/L)	8.58 ± 0.35	8.63 ± 0.39	10.19 ± 0.45 * C	7.16 ± 0.63	8.16–9.98
HGB (g/dL)	15.29 ± 0.58	15.17 ± 0.85	18.47 ± 0.69	12.75 ± 0.97 * C	12.4–15.4
HCT (%)	42.80 ± 1.60	42.83 ± 2.78	55.86 ± 2.16	37.30 ± 2.40	43.5–55.4
MCV (fL)	50.01 ± 0.47	50.77 ± 0.41	55.07 ± 0.26	52.37 ± 1.48	50.8–55.6
MCH (pg)	17.76 ± 0.13	17.71 ± 0.17	18.10 ± 0.15	17.88 ± 0.35	13–15.5

WBC—white blood cells, Ly—lymphocytes, Mo—monocytes, Gran granulocytes, RBC—red blood cells, HGB—hemoglobin, HCT—hematocrit, MCV—mean corpuscular volume, MCH—mean corpuscular hemoglobin. Control C (n = 18), AM1 (DSS 1%; n = 18), AM3 (DSS 3%; n = 18), AM5 (DSS 5%; n = 18). The results are presented as means ± SD. * p < 0.05, ** p < 0.01, *** p < 0.001.

**Table 5:** Intestinal morphology of the PGF mice following 5-day exposure to DSS.

Group	Cross-Section of Villi (µm^2^)	Villus Perimeter (µm)	Villus Height (µm)	Crypt Depth (µm)	Ratio Villus Height/Crypt Depth
Control C	121,300 ± 4089	1165 ± 27.10	430.5 ± 10.27	120.2 ± 2.46	3.624 ± 0.11
AM1	93,450 ± 997 *** C	1124 ± 22.06	406.6 ± 6.26	111.1 ± 1.30 ** C	3.664 ± 0.06
AM3	92,620 ± 1091 *** C	1080 ± 23.84	371.9 ± 7.67 *** C	103.3 ± 1.18 * AM1 *** C	3.622 ± 0.09
AM5	79,840 ± 3980 ** AM3 *** AM1, C	811 ± 32.20 *** AM1, AM3, C	307.7 ± 11.26 *** AM1, AM3, C	70.90 ± 2.02 *** AM1, AM3, C	4.425 ± 0.20 *** AM1, AM3, C

Control C (n = 18), AM1 (1% DSS, n = 18), AM3 (3% DSS, n = 18), AM5 (5% DSS, n = 18). DSS dextran sodium sulphate, PGF pseudo germ-free mice. The results are expressed as means ± SD. * *p* < 0.05, ** *p* < 0.01, *** *p* < 0.001.
